# Supplementary figures and images for: The neurocognitive role of working memory load when Pavlovian motivational control affects instrumental learning
Source: PLoS Comput Biol. 2023 Dec 8;19(12):e1011692. doi: 10.1371/journal.pcbi.1011692 (PMC10732416; doi:10.1371/journal.pcbi.1011692)

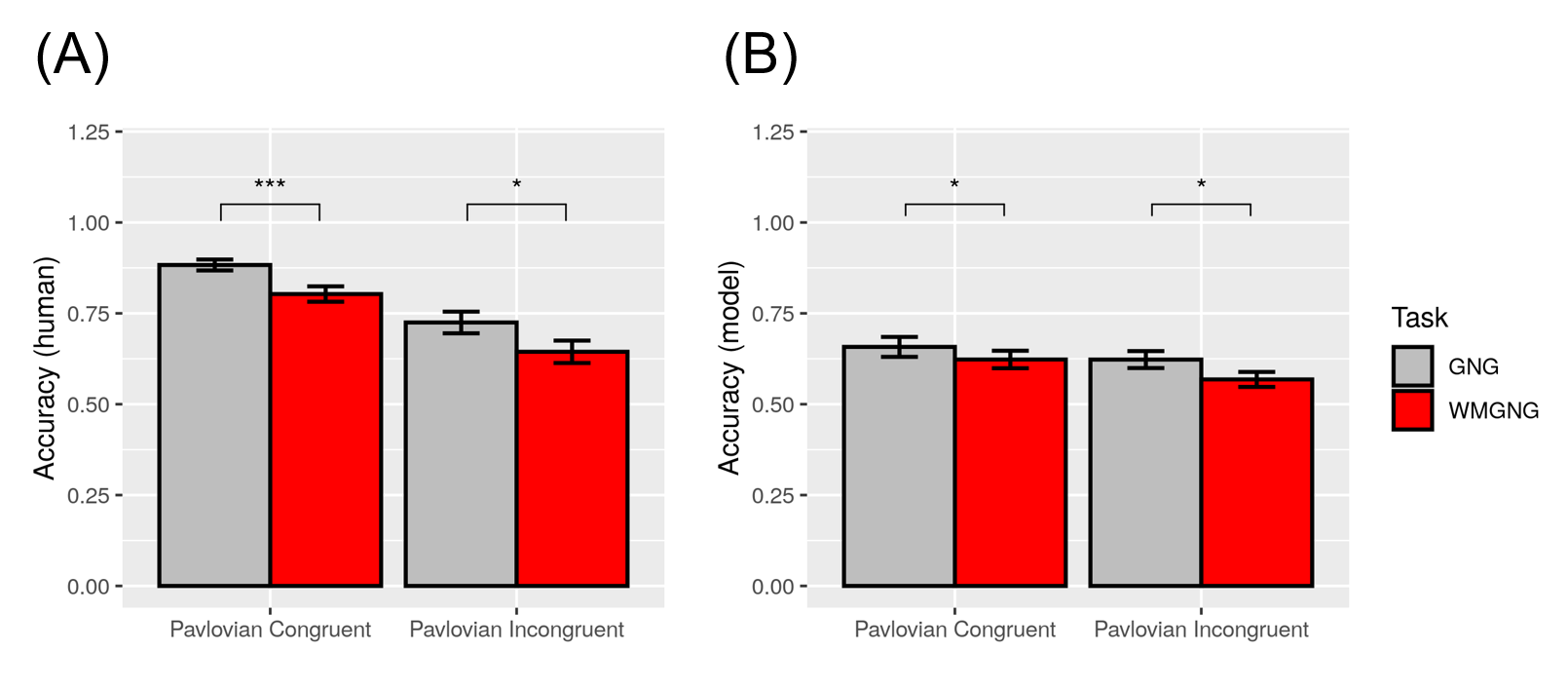

Supplement: S1 Fig — (A) Participants’ accuracy in the Pavlovian-congruent and Pavlovian-incongruent conditions, separately for the GNG and WMGNG tasks. Participants showed lower accuracy in the WMGNG compared to the GNG task both in the Pavlovian-congruent and Pavlovian-incongruent conditions. (B) Prediction from the best model regarding the accuracy in the Pavlovian-congruent and Pavlovian-incongruent conditions. We used a one-step ahead prediction for generating the model predictions. In line with our participants’ data, our model predictions showed lower accuracy in the WMGNG than the GNG task in both types of conditions. In all figures, error bars indicate means ± standard errors of the means. (PNG) [file pcbi.1011692.s003.png]

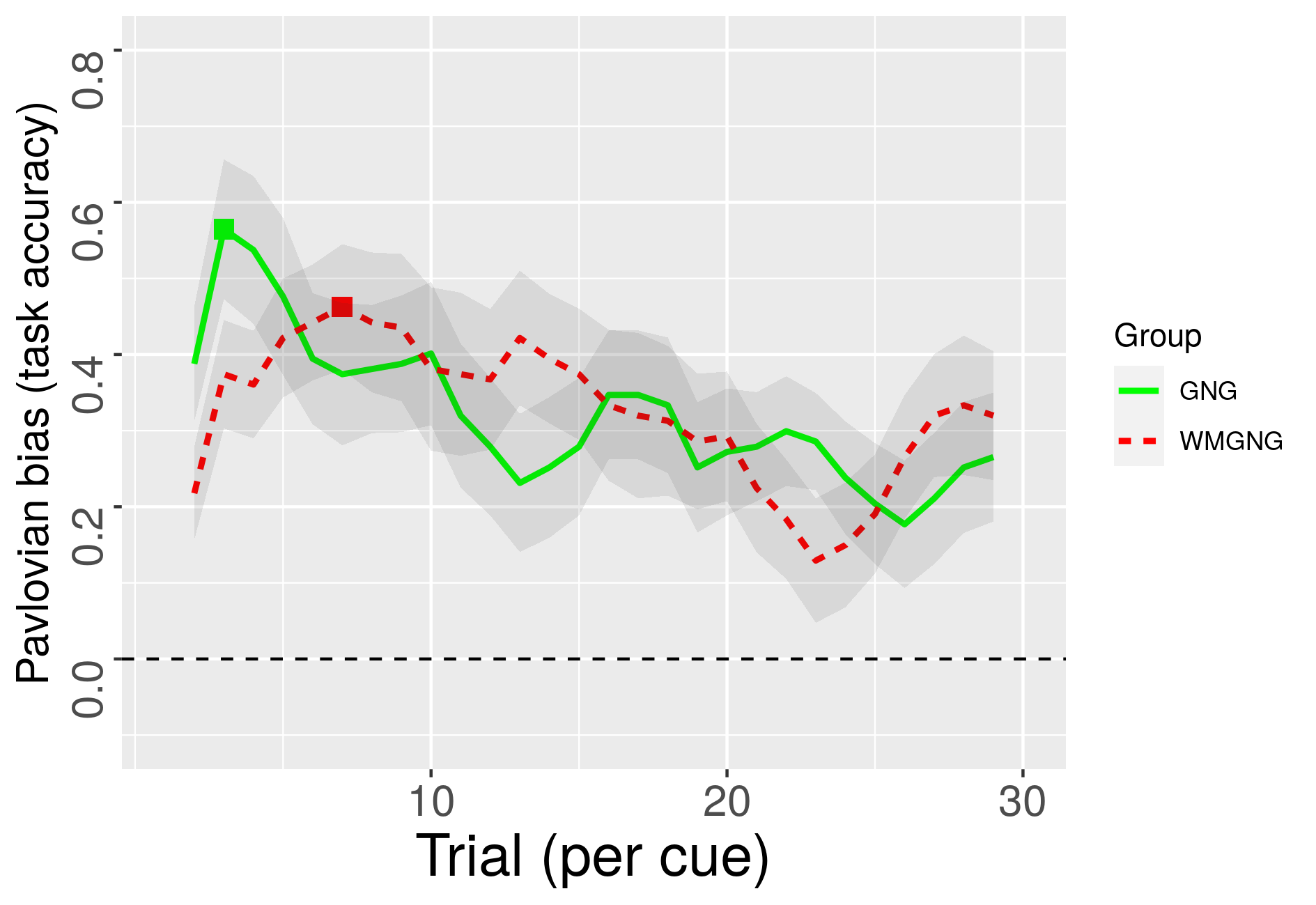

Supplement: S2 Fig — The behavioral measure of Pavlovian bias, which is the difference in accuracy between the Pavlovian-congruent and Pavlovian-incongruent conditions, plotted as a function of trial separately for the GNG and WMGNG tasks. While the peak in the WMGNG task appeared later than the GNG task, Pavlovian bias was similar between the tasks after the peaks. Moving average smoothing was applied with filter size 3. Lines indicate group means and ribbons indicate means ± standard errors of the means. Squares indicate the peak values of Pavlovian bias during the two tasks. (PNG) [file pcbi.1011692.s004.png]

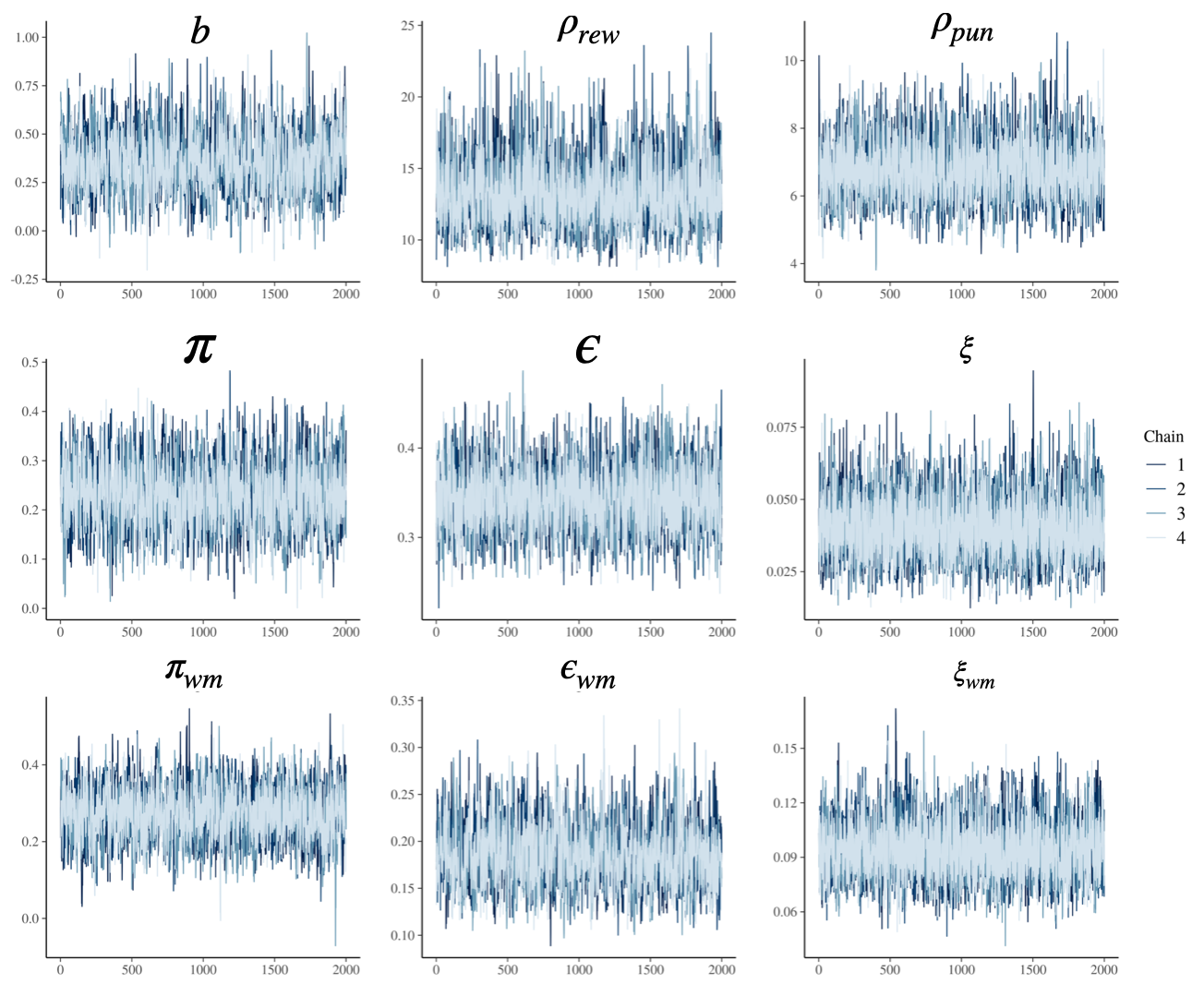

Supplement: S3 Fig — The trace plots show that MCMC samples were well mixed and converged. Note that the plots excluded burn-in samples. (PNG) [file pcbi.1011692.s005.png]

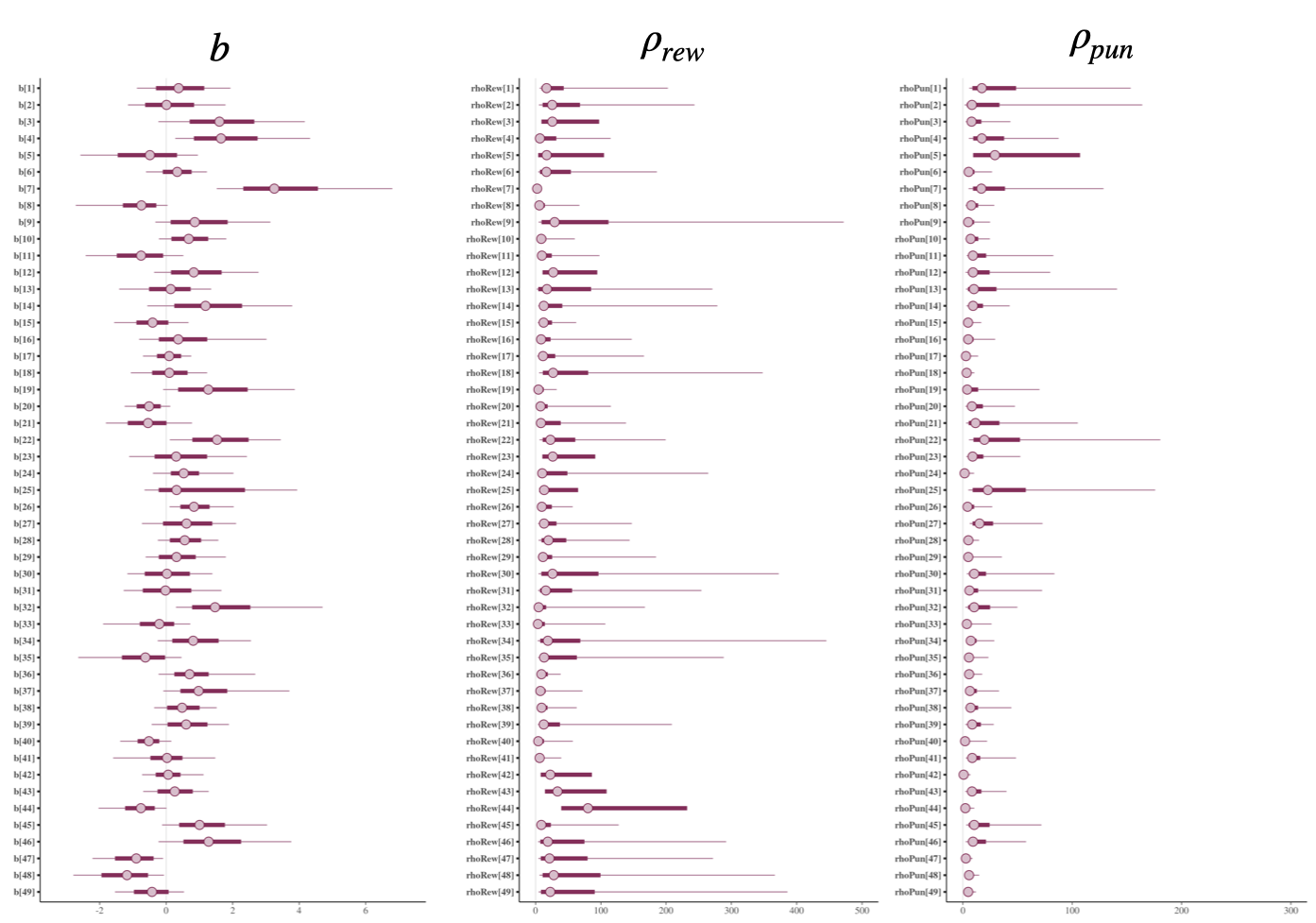

Supplement: S4 Fig — Dots indicate medians and thick bars indicate 95% HDIs. (PNG) [file pcbi.1011692.s006.png]

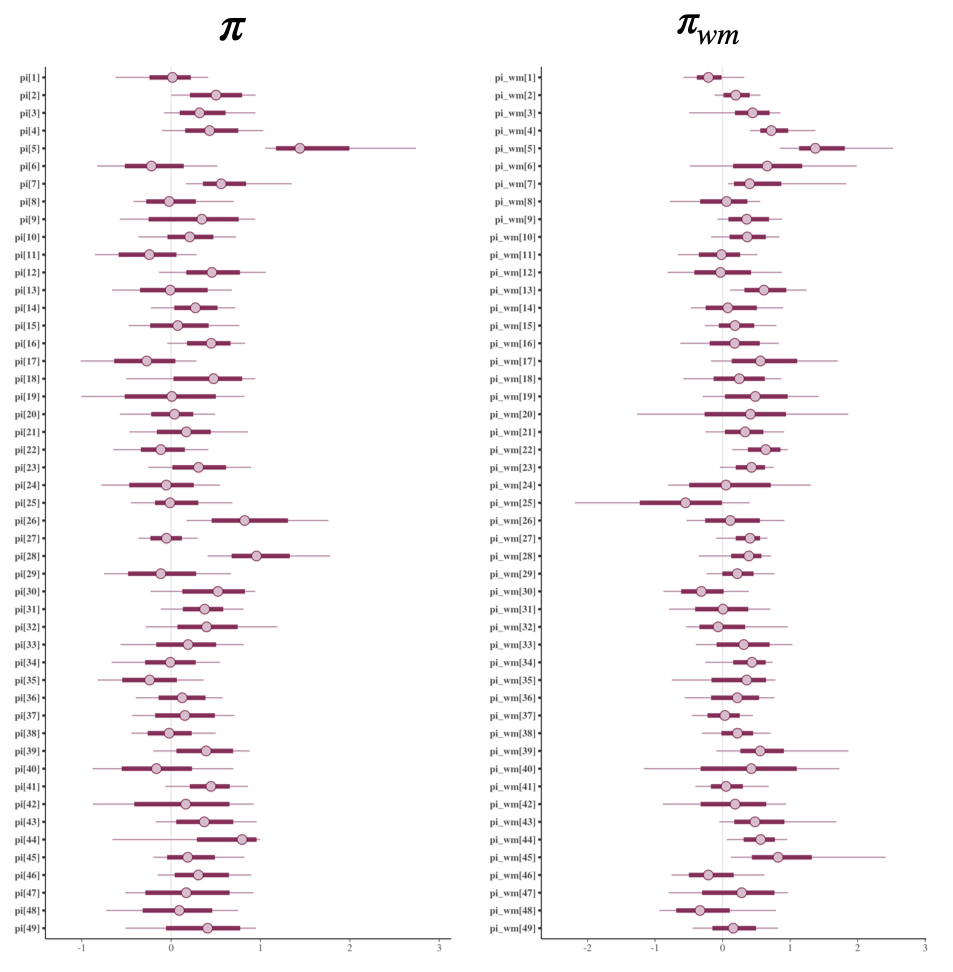

Supplement: S5 Fig — Dots indicate medians and thick bars indicate 95% HDIs. (PNG) [file pcbi.1011692.s007.png]

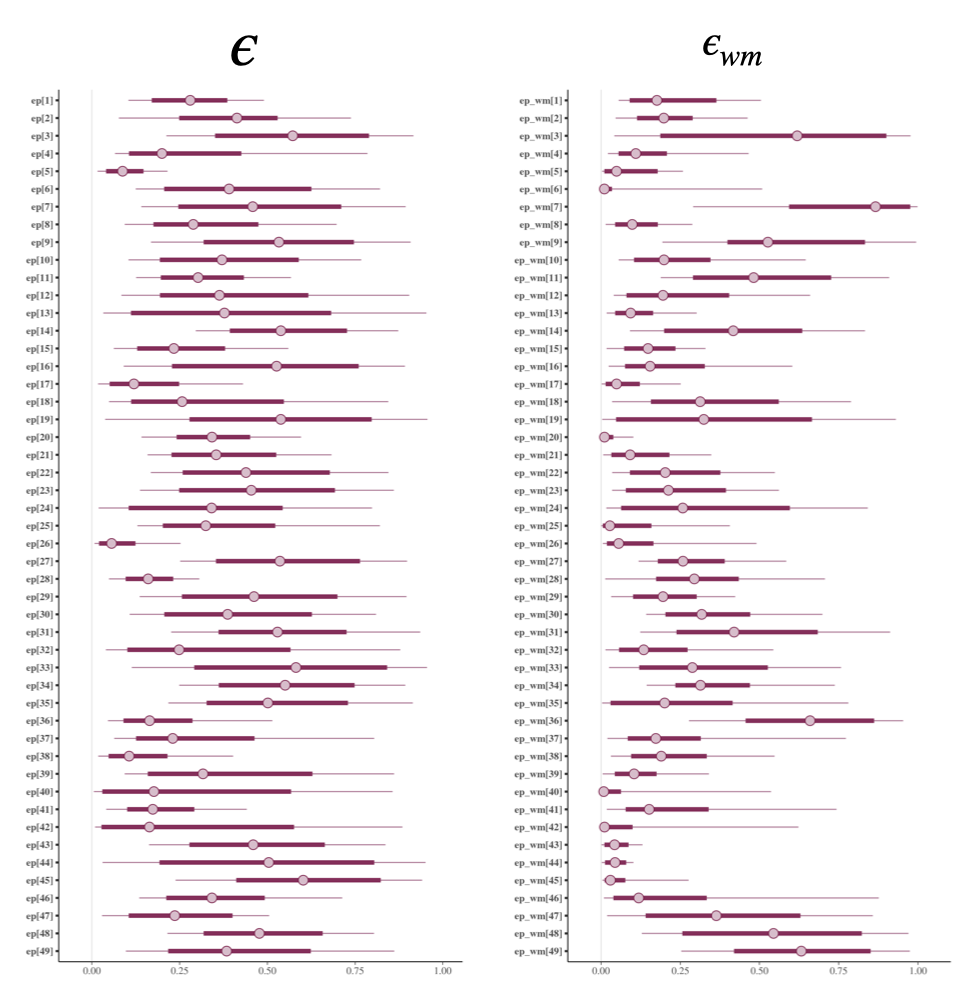

Supplement: S6 Fig — Dots indicate medians and thick bars indicate 95% HDIs. (PNG) [file pcbi.1011692.s008.png]

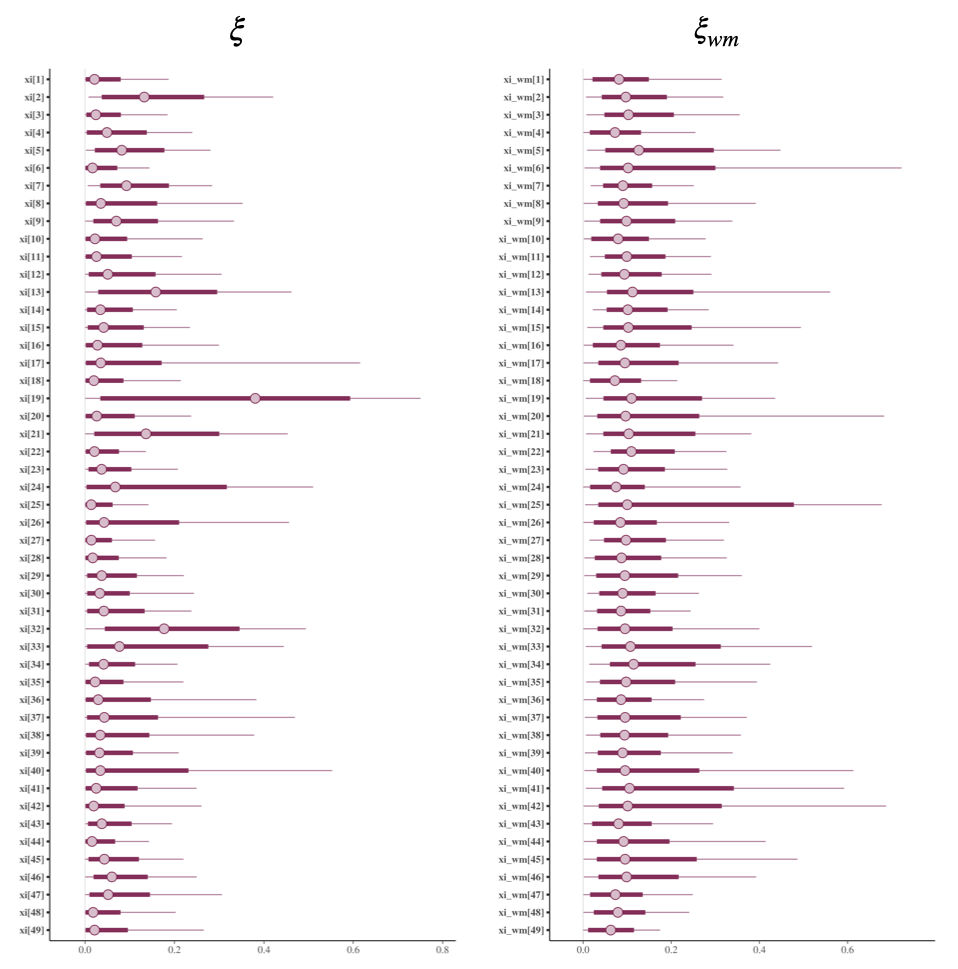

Supplement: S7 Fig — Dots indicate medians and thick bars indicate 95% HDIs. (PNG) [file pcbi.1011692.s009.png]

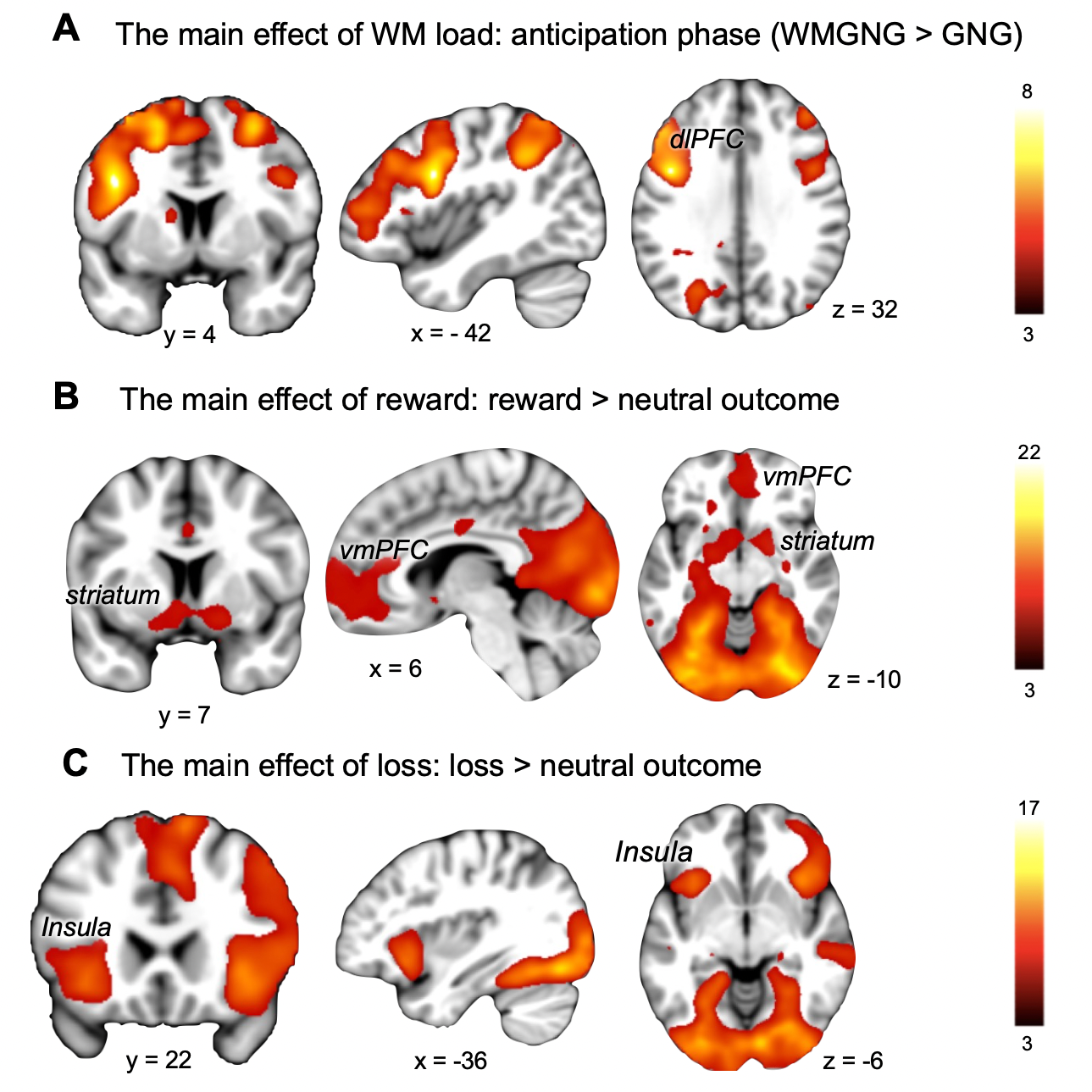

Supplement: S8 Fig — (A) The main effect of WM load during the anticipation phase. As reported in previous meta-analysis studies [29,129], under WM load, regions of the lateral prefrontal cortex (PFC), including bilateral superior gyrus (MNI space coordinates x = -22, y = 0, z = 50, Z = 6.03, p < 0.05 whole-brain cluster-level family-wise error (FWE)) and middle frontal gyrus (MNI space coordinates x = 29, y = 2, z = 57, Z = 5.44, p < 0.05 whole-brain cluster-level FWE), and left precentral gyrus (MNI space coordinates x = -45, y = 5, z = 30, Z = 5.79, p < 0.05 whole-brain cluster-level FWE) and left inferior parietal cortex (MNI space coordinates x = -38, y = -51, z = 41, Z = 5.42, p < 0.05 whole-brain cluster-level FWE) showed increased BOLD signal. These results indicate that participants indeed had cognitive loads in the brain level. (B) The main effect of reward outcome. The reward was significantly associated with the signal in the striatum (MNI space coordinates x = 18, y = 5, z = -12, Z = 4.47, p < 0.05 whole-brain cluster-level FWE) and ventromedial PFC (MNI space coordinates x = -3, y = 68, z = 4, Z = 4.92, p < 0.05 whole-brain cluster-level FWE). (C) The main effect of loss outcome. Loss-related regions such as the insula (MNI space coordinates x = -36, y = 19, z = -10, Z = 6.77, p < 0.05 whole-brain cluster-level FWE) showed increased BOLD response. (B) and (C) are consistent with the previous findings suggesting reward- and loss-related regions [130,131]. Overlays are shown with a threshold of p < 0.001 (uncorrected). Color scale indicates t-values. (PNG) [file pcbi.1011692.s010.png]
