# Supplementary material for: The neurocognitive role of working memory load when Pavlovian motivational control affects instrumental learning
Source: PLoS Comput Biol. 2023 Dec 8;19(12):e1011692. doi: 10.1371/journal.pcbi.1011692 (PMC10732416; doi:10.1371/journal.pcbi.1011692)
Supplement: Attachment — Submitted filename: response_to_reviews_final.pdf [file pcbi.1011692.s017.pdf]

Dear Editor,

We thank the editor and reviewers for their insightful and constructive comments, and for allowing us to revise our manuscript in response to the critiques.

In the subsequent pages, we detail our point-by-point responses to the reviewers' comments. We have addressed most of the issues raised by the reviewers. The reviewers' comments are in blue (and bolded) and our responses are in black.

We hope that you will now find the improved version of the manuscript ready for publication in *PLOS Computational Biology*, and we look forward to your response soon.

Sincerely,
Woo-Young Ahn

Reviewer #1

C1) WMload would not only affect the learning of the correct instrumental response to make (go/no-go) but also learning about which outcome is signalled by the cue (potential reward or punishment). The rate of learning about the cue-outcome relationships directly affects the expression of Pavlovian biases and thus these biases could be expected to emerge more slowly in those who are under WM load compared to those who are not. However, this nuance is not captured in the current analyses where the order of tasks is counterbalanced and collapsed (because task order is not a parameter in the computational model). I think a stronger test of the hypotheses would be the comparison of Pavlovian biases at the end of task 1 vs. beginning of task 2 for the subset of participants who did the tasks in the order GNG then WMGNG. Does WM load lead to an immediate increase in Pavlovian bias? Another option would be to first ensure no difference in Pavlovian biases at the end of task 1 between the two groups. Even if the biases emerged more slowly in the WMGNG group, they may still be comparable at the end of the first task. Differences in performance could then be examined between the two groups during task 2. This would directly address the question of "once these Pavlovian biases have been learned, does WM load increase the expression of them"? I realise that the study was not powered for between group analyses, but if the authors wish to make the claim that WM load does not affect the expression of Pavlovian bias then the effects of WM load on cue-outcome learning vs. bias expression need to be separated out. It seems that a between groups design would have been better to test these hypotheses (where all participants are first given the opportunity to learn the relationships without WM load and then half of them are placed under WM load for the second half). A between-groups design was indeed used by Otto to examine the balance of model-free/model-based learning which the authors refer to repeatedly. The authors should discuss the current design as a limitation and perhaps tone down the interpretation of the current findings unless I have missed something critical?

Response:

We thank the reviewer for pointing it out. We agree that cue-outcome learning and Pavlovian bias need to be separated out. To address this issue, the reviewer suggested treating task 1 as cue-outcome learning phase and analyzing the Pavlovian bias only using the data from task 2. However, this raises another issue because the fractal cues in task 2 were different from the fractal cues in task 1. Participants had to learn new cue-outcome relationships in task 2. We noticed that we did not include this detail in our

previous manuscript, so we added it in Materials and Methods. We apologize for the omission of an important detail.

To address the issue in a different way, we made a plot showing how the Pavlovian bias (accuracy difference between Pavlovian congruent and incongruent conditions) develops over time (**Fig S2**). In line with the reviewer's prediction, the peak Pavlovian bias appeared more slowly in the WMGNG than GNG task. However, after the peak, the Pavlovian bias was comparable between the two tasks. This might suggest that the expression of Pavlovian bias is similar between the two tasks after the cue-outcome association has been learned. We are aware that this is not a complete account of the issue and added a paragraph to discuss the limitations of our study design.

We added a supplementary figure (**Fig S2**) and made the following changes to our manuscript:

Page 9:

"However, participants could have been slower in learning the Pavlovian cue-outcome associations under WM load, as the instrumental learning became slower under WM load. Thus, it should be examined if participants expressed similar levels of Pavlovian bias in the GNG and WMGNG tasks after they learned the cue-outcome associations in both tasks. To this end, we plotted the temporal development of Pavlovian bias across trials (**Fig S2**). We observed a delayed peak in the WMGNG compared to the GNG task, which indicates that acquiring Pavlovian associations could have taken more time under WM load. Nonetheless, we observed similar levels of Pavlovian bias in both tasks after the initial peak. Thus, we concluded that our data do not show noticeable changes in Pavlovian bias under WM load."

Page 22:

"One limitation of our study design is that the acquisition of Pavlovian cue-outcome associations and the expression of Pavlovian bias are not clearly separated. A more sophisticated way of comparing Pavlovian bias would first guarantee that participants acquired the Pavlovian associations to similar degrees in both tasks. Although we conducted a similar analysis (**Fig S2**), it is not a complete solution to the issue because we cannot separate the two components with the data from our task. Future studies can address this issue by using a between-subject design, where the WM load are applied to only one group of participants after all participants perform enough trials of the GNG task. Another way would be to use the Pavlovian-instrumental transfer paradigm, which is similar to our paradigm but separates the Pavlovian learning, instrumental learning,

and Pavlovian-instrumental transfer phases. Experimenters can manipulate the WM load during the transfer phase to examine the WM load effect on Pavlovian bias.”

Page 25:

“The order of task completion was counterbalanced among participants; some participants performed the GNG task first while others began with the WMGNG task.”

“We used different sets of fractal stimuli for the practice, first, and second tasks.”

Page 34:

“**Fig S2. Temporal development of Pavlovian bias (N=49).** The behavioral measure of Pavlovian bias, which is the difference in accuracy between the Pavlovian-congruent and Pavlovian-incongruent conditions, plotted as a function of trial separately for the GNG and WMGNG tasks. While the peak in the WMGNG task appeared later than the GNG task, Pavlovian bias was similar between the tasks after the peaks. Moving average smoothing was applied with filter size 3. Lines indicate group means and ribbons indicate means ± standard errors of the means. Squares indicate the peak values of Pavlovian bias during the two tasks.”

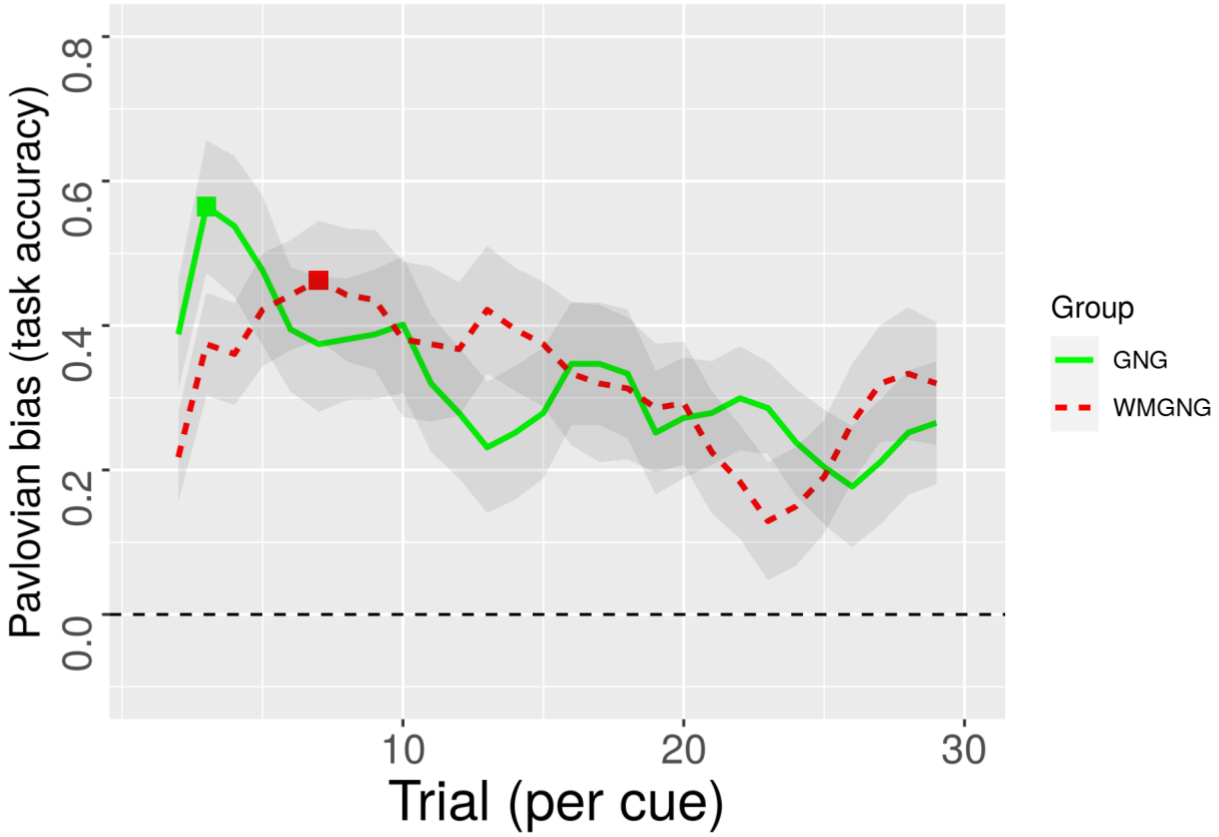

C2) If we do believe that WM load does not affect the expression of Pavlovian biases then what does this mean for our clinical models? As the authors mention, numerous studies have demonstrated that the Pavlovian biases are stronger in those suffering from various clinical disorders. Deficits in executive control are often identified as the culprit here, but the results of this study (if true) would suggest instead that reward and punishment are more motivationally salient in these groups and that executive control deficits are largely irrelevant. This is a very interesting conclusion which I think could be explored a little more in the discussion.

Response:

We appreciate the reviewer for suggesting an important discussion point. We have added the following paragraph in the discussion.

Page 21:

"Our result help refine the understanding of elevated Pavlovian bias in individuals suffering from certain mental disorders [48,49,96]. Researchers often assume the increased Pavlovian bias in clinical populations is, at least in part, a consequence of their impaired executive control, which hinders their goal-directed behavior [47,51]. However, the causal relationship between executive control and the Pavlovian influence on behavior has not been actively investigated. In this study, we showed that constraining executive control with WM load did not increase the Pavlovian bias. It suggests that executive control deficits observed in clinical populations may not directly contribute to their heightened Pavlovian bias. Instead, clinical populations might perceive reward or punishment (or both) as more motivationally salient than healthy individuals, which leads to stronger urges for Pavlovian responses. Nonetheless, one should be cautious in this interpretation because the executive control impairments in clinical populations might be more severe than (or qualitatively different from) the ones elicited with WM load in our study."

Reviewer #2

C1) I found the term "Pavlovian influence" confusing because there are two pavlovian effects: the reward/go effect and the effects on task learning. The former is not really Pavlovian as much as it is a motivational congruency effect. I am not attached to specific terms here, it is just very confusing to track these separate ideas throughout the paper.

Response:

As the reviewer pointed out, the tendencies to give a go response toward a reward and a no-go response from a punishment are regarded hard-wired (or unconditioned responses, in the Pavlovian conditioning terms). In our task, reward and punishment forms Pavlovian associations with the fractal cues we use for instrumental learning. Thus, the fractal cues incidentally elicit go or no-go as conditioned responses regardless of instrumental requirements. Our assumption is that this acquisition of Pavlovian conditioned responses occurs in parallel with instrumental learning, and that a participant's response for each trial is determined by integrating the Pavlovian conditioned response and the instrumentally trained response. This corresponds to the $W(a_t, s_t)$ calculation in our model. The performance in our learning task increases when the two responses are congruent (e.g., both preferring go), and decreases when they are incongruent (e.g., one preferring go and the other preferring no-go).

We do not assume that the Pavlovian component in our task influences the instrumental action value updating. For example, we do not assume that the learning rates are different between the Pavlovian congruent and incongruent conditions (e.g., de Boer et al., 2019; Swart et al., 2017). Instead, we assume that the Pavlovian conditioned responses are input to the action selection state together with the instrumentally trained responses (see above). This approach allows researchers to investigate the balance between the two systems in animal and human behavior (Dayan et al., 2006; Dorfman & Gershman, 2019; Guitart-Masip et al., 2012).

To clarify how Pavlovian bias is conceptualized in our paper, we made the following changes to our manuscript:

Page 5:

"In a widely-used paradigm, Pavlovian bias emerges when the cues used in a instrumental learning task trigger Pavlovian conditioned responses (e.g., active responses to reward cues regardless of action requirements), leading to an increase or

decrease in task performance [45,46]. Many computational models of the phenomenon assume that decision-makers consider both instrumentally trained responses and Pavlovian conditioned responses when deciding which response to give in each trial. This decision-making procedure balances the instrumental and Pavlovian learning systems, where the instrumental system is more flexible but computationally more expensive while the Pavlovian system is more rigid but computationally cheaper. This is because the instrumental system learns stimulus-response-outcome associations, while the Pavlovian system only learns stimulus-outcome associations and has hard-wired responses for the learned associations."

Page 27:

"A value of π close to 0 means that the agent relies mostly on the instrumental system (and the go bias), while a larger value of π means that the Pavlovian system also has a great impact on the agent's action selection."

C2) The authors refer to lower learning rate as "slower learning" several times. This is common in the field, but is not really accurate, and in a way that is important for this paper: lower learning rates integrate over longer time horizons than faster learning rates. This has a tradeoffs (i.e., noise has less of an influence, better memory, but slower to recognize shifts in reward structure). There are no shifts in reward structure here, so lower learning rates are primarily adaptive in the sense that they can compensate for the short memory capacity of WM. The authors should give more attention to the subtlety in this parameter. This is particularly relevant to the finding that behavioral learning rates were reduced whereas striatal correlates of learning were increased under WM load.

Response:

We thank the reviewer for pointing it out. We acknowledge that we need to change some expressions regarding the term. As far as we understood, the reviewer's concern is that the expression 'slower learning' might be interpreted by readers as maladaptive or impaired learning. Thus, we changed the wordings that explicitly or implicitly implies any quality judgment about lower learning rates.

As the reviewer pointed out (and as we mentioned in the manuscript), fast and slow learning have both pros and cons, and neither one is always better than the other (Collins, 2018). Also, explaining learning rate as learning speed is a useful metaphor because higher learning rates lead to more agile and responsive value updating toward the feedback in each trial than lower learning rates. Thus, we tried to fix our expressions appropriately in each sentence instead of changing all 'slower learning' to 'lower learning rates'.

Here are the changes we made to the manuscript:

Page 2:

“Behavioral and computational modeling analyses revealed that WM load reduced the learning rate and increased random choice, without affecting Pavlovian bias.”

“These results suggest that the limitation of cognitive resources by WM load promotes slow and incremental learning through the weakened cooperation between WM and RL”

Page 14:

“While the accuracy decrease could also be associated with the lower learning rate under WM load, we need to be cautious in this interpretation because lower learning rates can rather increase the accuracy by making learning more robust against noise.”

Page 18:

“In particular, our behavioral analysis revealed a shallower learning curve under WM load (**Fig 2C**).”

“Such a shift toward slow, incremental learning can be adaptive because it is more reliable under resource-limited conditions and robust against noise in stationary environments [69].”

Page 20:

“Instead of relying more on the Pavlovian system, which might only provide little boost in resource efficiency in our task, the participants may simply have favored slow, incremental learning, which requires small cognitive resources.”

C3) The above-two points combined lead to much confusion and make me disagree with some key assertions in the paper: For example, from the abstract, the authors say: “These results suggest that the limitation of cognitive resources by WM load decelerates instrumental learning through the weakened cooperation between WM and RL; such limitation also makes action selection more random, but it does not directly affect the balance between instrumental and Pavlovian systems.” What does it mean for instrumental learning to decelerate? The model does not test the balance between the systems, and that finding is based on the go-congruency effect (which is unrelated to instrumental learning, at least in their model). If anything, there is evidence for a change in the balance of the systems from the PPI results.

Response:

We thank the reviewer for pointing it out and giving us an opportunity to clarify it. As we mentioned in C1, we conceptualize the Pavlovian system as a separate valuation system that ‘competes with’ the instrumental system for action selection in our instrumental learning task. In this framework, Pavlovian bias indicates how much impact the Pavlovian system has on action selection during the task. The Pavlovian bias parameter (π) in our model represents the magnitude of such impact and thus reflects the balance between the instrumental and Pavlovian values in action selection. A value of π close to 0 means that the agent relies mostly on instrumental values (and the go bias), while a larger value of π means that Pavlovian values also have a great impact on the agent’s action selection. We concluded that the balance between the instrumental and Pavlovian systems did not change under WM load because the Pavlovian bias parameter without WM load (π) was not credibly different from the one with WM load (π_{wm}). Also, the behavioral measure of Pavlovian bias was not different between the GNG and WMGNG tasks. In addition, the PPI analysis aimed to test if the interaction between the prefrontal WM and striatal RL systems changes under WM load, not the interaction between Pavlovian and instrumental systems. While some researchers proposed the prefrontal cortex could be important for mitigating the Pavlovian influence on behavior, there has been no direct evidence to our knowledge that corticostriatal connectivity can be a measure of the balance between the Pavlovian and instrumental systems. In another fMRI analysis in the paper, we tested if WM load alters the brain activation associated with Pavlovian bias and we did not find any region with significantly different activation related to Pavlovian bias under WM load. Thus, we concluded that WM load did not change the balance between the Pavlovian and instrumental systems. We hope this explanation clarifies our points.

In addition, we made the following revision to the abstract to elaborate on what we mean by “deceleration”:

Page 2:

“These results suggest that the limitation of cognitive resources by WM load promotes slow and incremental learning through the weakened cooperation between WM and RL”

C4) For the PPI results, the authors should plot the baseline connectivity values (perhaps from ROIs). If the baseline connectivity is negative, then the result would indicate increased negative connectivity, which would have a different interpretation.

<u>Response</u>:

We thank the reviewer for pointing it out. To examine whether the functional connectivity between the striatum and the prefrontal cortex during the anticipation phase was positive, we calculated the mean beta estimate for each condition or task. Our results indicated that functional connectivity between the striatum and dlPFC/vmPFC was positive both in the GNG and WMGNG tasks, with lower connectivity in the WMGNG than the GNG task (**Fig 5B**).

We added beta plots to **Fig 5B** and made the following changes to the manuscript:

Fig 5. fMRI results (N=44). (A) In the predefined ROI-based analysis, RPE signaling in the striatum was stronger in the WMGNG task than in the GNG task. The left figure shows significant regions at p < 0.05 (SVC) in yellow. The right figure shows an increased mean beta estimate in the striatum under WM load. (B) The left figure shows that functional connectivity between the striatum (seed region, top) and prefrontal regions, including vmPFC (bottom left) and dlPFC (bottom right), was weaker in the WMGNG task than in the GNG task when computing reward expectation ($p < 0.05$, whole-brain cluster-level FWE). The right figures show decreased mean beta estimates in vmPFC and dlPFC under WM load. In all figures, error bars are means ± standard errors of the means. Overlays are shown with a threshold of $p < 0.001$ (uncorrected). Color scale indicates t-values.

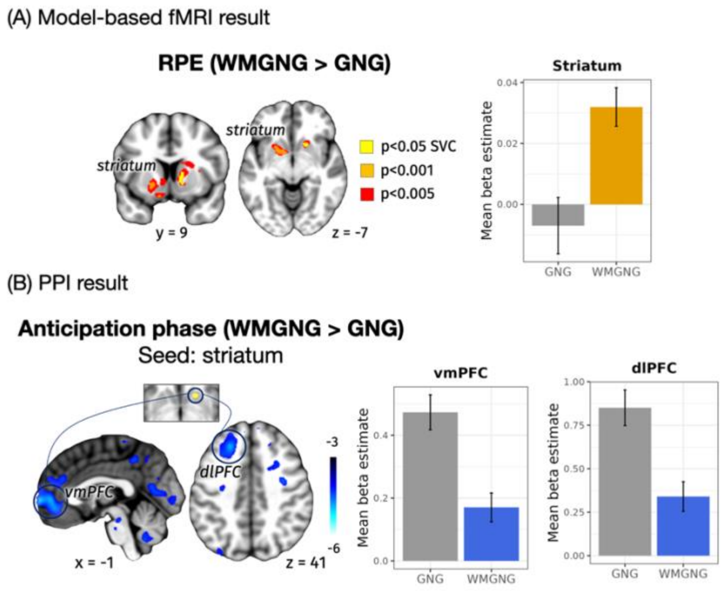

Page 17:

“The right figures show decreased mean beta estimates in vmPFC and dlPFC under WM load. In all figures, error bars are means ± standard errors of the means.”

C5) The authors should be more cautious about reverse inference when referring to the striatal prediction error signal.

Response:

We thank the reviewer for pointing it out and have made following changes to the manuscript to avoid reverse inference when interpreting the RPE and other neuroimaging results:

Page 6:

“We observed increased striatal RPE signaling which was in line with our hypothesis that the contribution of RL will increase under WM load.”

Page 18:

“These findings support theoretical explanations that WM load reduces the contribution of the WM system and increases the contribution of the RL system.”

Page 19:

“These ideas are further supported by our finding that the striatum showed weakened functional connectivity with the dlPFC during reward prediction under WM load (**Fig 5A**). This result is in line with theoretical predictions (1) that the WM system would become unreliable and thus its contribution to instrumental learning would decrease and (2) that that the reward prediction information delivered from the WM to the RL system would become less precise and the RL system would rely less on it.”

“Future research is needed to clarify which function of vmPFC was influenced by our WM load manipulation.”

In addition, we deleted some sentences and slightly changed the structure of paragraphs. For more detail, please refer to pages 18 and 19 in the manuscript with tracked changes.

C6) The main test is missing detail about the whole-brain and SVC correction procedures

Response:

We thank the reviewer for pointing it out. In the revised version, we have added a section called "Corrections for multiple comparisons" in Page 33, which is included below:

Page 33:

Corrections for multiple comparisons

For each GLM, we performed whole-brain FWE corrections at the cluster level (corrected $p < 0.05$, with the height threshold of $p < 0.001$) for multiple comparisons [127,128] using SPM12. For a priori ROIs, we used SVC using SPM12.

C7) Why is the olfactory bulb in the striatum ROI?

Response:

We apologize for our mistakes in the text about the striatum ROI. We included the "olfactory cortex", not the "olfactory bulb" in the striatum ROI. We included the olfactory cortex in the striatum ROI following the recommendation from a prior work using the AAL3 atlas (Rolls et al., 2020). In the AAL3 atlas, the olfactory cortex (OLF) includes the olfactory tubercle, which is a part of the ventral striatum.

We fixed the "olfactory bulb" into "olfactory cortex" in all the incorrectly written sentences.

Page 31:

(Line 1042) "The striatum ROI was constructed anatomically by combining the AAL3 definitions of bilateral caudate, putamen, olfactory cortex, and nucleus accumbens."

Page 32:

(Line 1068) "The striatum ROI was constructed anatomically by combining the AAL3 definitions of bilateral caudate, putamen, olfactory cortex, and nucleus accumbens."

C8) The authors use a different noise parameter than is common in the literature (softmax noise). I am curious for the authors to explain their rationale behind this choice.

Response:

We thank the reviewer for giving us an opportunity to explain the rationale behind our model component choices. We have made the following changes to the document to explain why we did not use the softmax inverse temperature parameter and why we used the irreducible noise parameter. Note that the original model for the task (Guitart-Masip et al., 2012, Neuroimage) also used the same form (i.e., using no inverse temperature parameter but including the noise parameter)

Page 28:

"We did not include the widely used inverse temperature parameter because ρ_{rew} and ρ_{pun} serve the same role as inverse temperature, deciding the level of determinism when generating actions based on action weights [56]. On the other hand, ξ represents the constant randomness regardless of action weights, and has been widely used by researchers using similar models to ours [45,56,64,112,113]."

C9) Why would participants perform worse when choices are easier in WM condition? That seems counterintuitive. Does the model predict this behavior?

Response:

In our view, WM load decreases the performance even in easier conditions because it increases the choice randomness.

Increase in randomness would lead to a decrease in accuracy in most cases unless the accuracy was below the chance level in the first place. For example, assume that a participant found our task very easy, so he has perfectly learned all the conditions in the task. If the participant is completely deterministic (i.e., 0% choice randomness), the participant will show 100% accuracy after he or she finished learning. However, if the choice randomness increases to 10%, the accuracy will decrease to 95% because he will be correct only half of the time when he chooses randomly (0.95 = 0.9 * 1.0 + 0.1 * 0.5). The increase in randomness under WM load could be associated with the difficulty in retaining various information in WM. Information including cue, reward expectation, and intended response needs to be in WM until response generation but some information might be lost because the 2-back task is using some of the WM capacity.

We performed a posterior predictive check analysis to check whether our model predicts this phenomenon and confirmed that it does.

We added our new figure (**Fig S1**) in the supplementary and made the following changes to our manuscript:

Page 8:

"Participants' performance decreased both in the Pavlovian-congruent ("go to win" and "no-go to avoid losing") and Pavlovian-incongruent ("no-go to win" and "go to avoid losing") conditions (**Fig S1A**)."

Page 13:

"Next, we examined if our model predicts the observed decrease in task performance under WM load both in the Pavlovian-congruent and Pavlovian-incongruent conditions. **Fig S1B** shows that our model indeed predicts the lower task performance in both types of conditions. This result is in line with our result that choice randomness increased under WM load. We can expect that an increase in randomness would result in a lower accuracy unless the accuracy was below the chance level in the first place. While the accuracy decrease could also be associated with the lower learning rate under WM load, we need to be cautious in this interpretation because lower learning rates might instead increase the accuracy by making learning more robust against noise."

Page 33:

Fig S1. Performance in the Pavlovian-congruent and Pavlovian-incongruent conditions (N=49). (A) Participants' accuracy in the Pavlovian-congruent and Pavlovian-incongruent conditions, separately for the GNG and WMGNG tasks. Participants showed lower accuracy in the WMGNG compared to the GNG task both in the Pavlovian-congruent and Pavlovian-incongruent conditions. (B) Prediction from the best model regarding the accuracy in the Pavlovian-congruent and Pavlovian-incongruent conditions. We used a one-step ahead prediction for generating the model predictions. In line with our participants' data, our model predictions showed lower accuracy in the WMGNG than the GNG task in both types of conditions. In all figures, error bars indicate means ± standard errors of the means.

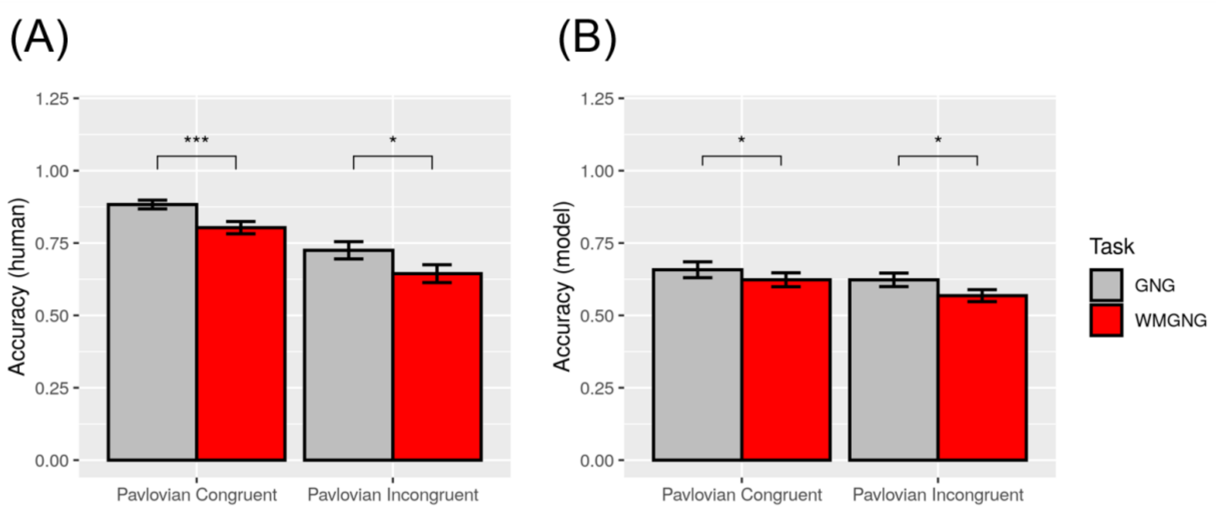

C10) I believe (though I could be mistaken) the authors need to submit their raw data to OpenNeuro or some other repository as well as more public info on the neuroimaging code in order to be compliant with journal policies. The neurovault maps are not enough to be able to reproduce the analyses.

Response:

We have shared the raw neuroimaging data in OpenNeuro (https://openneuro.org/datasets/ds004647/versions/1.0.2) and uploaded the analysis codes in Github (https://github.com/CCS-Lab/project_WMDM_public) so that readers could reproduce our neuroimaging results.

C11) Keeping track of the hypotheses by number is confusing. It would be better to repeat the hypotheses

Response:

We agree with the reviewer and edited the sentences where we refer to our hypotheses with numbers.

Page 9:

“Next, we tested the hypothesis that WM load would decrease the learning speed (**Fig 2C**).”

“To test the hypothesis that WM load would increase Pavlovian bias (**Fig 2D**), we quantified Pavlovian bias by subtracting the accuracy in Pavlovian-incongruent conditions (“no-go to win” and “go to avoid losing”) from the accuracy in Pavlovian-congruent conditions (“go to win” and “no-go to avoid losing”).”

Page 11:

“Importantly, the use of computational modeling allowed us to test our hypothesis that WM load would increase random choices; this would have not been possible if we had performed behavioral analysis alone.”

Page 12:

“**Fig 3B** illustrates that Pavlovian bias was not credibly different between the two tasks, consistent with our behavioral results that failed to show a change in Pavlovian bias under WM load.”

“These results support our hypotheses that WM load would reduce learning rate and that it would increase random choices.”

Page 15:

“This supports our hypothesis that WM load would increase the contribution of the RL system and decrease the contribution of the WM system.”

Page 16:

“We also tested the hypothesis that WM load would lead to a greater brain activation associated with Pavlovian bias, but we found no main effect of Pavlovian bias between the GNG and WMGNG tasks (WMGNG > GNG [Pavlovian-congruent > Pavlovian-incongruent]) within the striatum or SN/VTA ($p < 0.05$ SVC, a priori ROIs).”

“As a test of our hypothesis that WM load would increase choice randomness, we examined whether the WM load led to less brain activation associated with value comparison (which would indicate a decreased contribution of the value comparison signal and thus relatively more contribution of randomness).”

Page 31:

“GLM1 was used to test the hypothesis that WM load would increase the contribution of the slow, incremental RL system because the resources for the fast WM system are constrained.”

“GLM2 was used to test the hypothesis that neural responses associated with Pavlovian bias would increase under WM load.”

Page 32:

“GLM3 was used to the hypothesis that there would be less neural responses associated with value comparison signals under WM load.”

C12) What was the smoothing kernel (this should be in the main text)

Response:

As we mentioned in the “fMRI scans: general linear models” section (page 30), images were smoothed using a 3D Gaussian kernel (8mm FWHM).

C13) What was the multiband/acceleration protocol for the fMRI data?

Response:

We thank the reviewer for pointing it out. The multiband acceleration factor was 4 in the fMRI data. We added the information in the manuscript:

Page 30:

“Functional data was acquired using echo-planar imaging (EPI) in four scanning sessions containing 64 slices (TR = 1500ms, TE = 30ms, FOV = 256mm, 2.3mm × 2.3mm × 2.3mm, multiband acceleration factor = 4).”

C14) Several times the authors use “whole-based” when I think they mean “whole-brain”

Response:

We thank the reviewer for pointing out the typo and revised the manuscript accordingly.

Page 17:

“The left figure shows that functional connectivity between the striatum (seed region, top) and prefrontal regions, including vmPFC (bottom left) and dlPFC (bottom right),

was weaker in the WMGNG task than in the GNG task when computing reward expectation ($p < 0.05$, whole-brain cluster-level FWE)."

C15) Is the b parameter a go bias?

<u>Response</u>:

Yes, the b parameter represents a static bias of the individual to emit a 'go' response regardless of the learned values (Q and V). A positive b value indicates a constant bias toward the 'go' response, whereas a negative value represents a bias toward the 'no-go' response. While a more neutral description would be a 'static bias' parameter (Guitart-Masip et al., 2012) because the parameter can both indicate 'go' or 'no-go' biases, it is often denoted as a 'go bias' parameter even though negative values were allowed (Albrecht et al., 2016; Csifcsák et al., 2019; de Boer et al., 2019; Swart et al., 2017). We named it as a go bias following the convention of the literature.

References

Albrecht, M. A., Waltz, J. A., Cavanagh, J. F., Frank, M. J., & Gold, J. M. (2016). Reduction of Pavlovian Bias in Schizophrenia: Enhanced Effects in Clozapine-Administered Patients. *PloS One*, *11*(4), e0152781.

Collins, A. G. E. (2018). The Tortoise and the Hare: Interactions between Reinforcement Learning and Working Memory. *Journal of Cognitive Neuroscience*, *30*(10), 1422–1432.

Csifcsák, G., Melsæter, E., & Mittner, M. (2019). Intermittent Absence of Control during Reinforcement Learning Interferes with Pavlovian Bias in Action Selection. *Journal of Cognitive Neuroscience*, 1–18.

Dayan, P., Niv, Y., Seymour, B., & Daw, N. D. (2006). The misbehavior of value and the discipline of the will. *Neural Networks: The Official Journal of the International Neural Network Society*, *19*(8), 1153–1160.

de Boer, L., Axelsson, J., Chowdhury, R., Riklund, K., Dolan, R. J., Nyberg, L., Backman, L., & Guitart-Masip, M. (2019). Dorsal striatal dopamine D1 receptor availability predicts an instrumental bias in action learning. *Proceedings of the National Academy of Sciences of the United States of America*, *116*(1), 261–270.

Dorfman, H. M., & Gershman, S. J. (2019). Controllability governs the balance between Pavlovian and instrumental action selection. *Nature Communications*, *10*(1), 5826.

Guitart-Masip, M., Huys, Q. J. M., Fuentemilla, L., Dayan, P., Duzel, E., & Dolan, R. J. (2012). Go and no-go learning in reward and punishment: Interactions between affect and effect. *NeuroImage*, *62*(1), 154–166.

Rolls, E. T., Huang, C.-C., Lin, C.-P., Feng, J., & Joliot, M. (2020). Automated anatomical labelling atlas 3. *NeuroImage*, *206*, 116189.

Swart, J. C., Frobose, M. I., Cook, J. L., Geurts, D. E., Frank, M. J., Cools, R., & den Ouden, H. E. (2017). Catecholaminergic challenge uncovers distinct Pavlovian and instrumental mechanisms of motivated (in)action. *ELife*, *6*. https://doi.org/10.7554/eLife.22169
